# A Protocol for Characterizing Comprehensive Two‐Dimensional Liquid Chromatography Systems

**DOI:** 10.1002/jssc.70434

**Published:** 2026-05-11

**Authors:** Megane K. Aebischer, Marie Pardon, Clémence Gadot, Katia Arena, Niklas Carstensen, Michael Laemmerhofer, Paola Dugo, Francesco Cacciola, Luigi Mondello, Morgan Sarrut, Deirdre Cabooter, Sabine Heinisch, Davy Guillarme

**Affiliations:** ^1^ School of Pharmaceutical Sciences University of Geneva Geneva Switzerland; ^2^ Institute of Pharmaceutical Sciences of Western Switzerland University of Geneva Geneva Switzerland; ^3^ Laboratory for Pharmaceutical Analysis, Department of Pharmaceutical and Pharmacological Sciences KU Leuven Leuven Belgium; ^4^ Laboratory for Molecular Biodiscovery, Department of Pharmaceutical and Pharmacological Sciences KU Leuven Leuven Belgium; ^5^ Syensqo Lyon Research and Innovation Center Lyon France; ^6^ Messina Institute of Technology c/o Department of Chemical, Biological, Pharmaceutical and Environmental Sciences University of Messina Messina Italy; ^7^ Institute of Pharmaceutical Sciences, Pharmaceutical (Bio‐)Analysis University of Tübingen Tübingen Germany; ^8^ Chromaleont s.r.l., c/o Department of Chemical, Biological, Pharmaceutical and Environmental Sciences University of Messina Messina Italy; ^9^ Institut des Sciences Analytiques Université De Lyon Lyon France

**Keywords:** 2D‐LC, bidimensional liquid chromatography, characterization, LC × LC, system dispersion

## Abstract

Comprehensive two‐dimensional liquid chromatography (LC × LC) is an effective strategy to increase peak capacity and improve separation performance for complex samples, offering a valuable alternative to conventional one‐dimensional liquid chromatography (1D‐LC). However, LC × LC method development and optimization remain challenging due to the interconnection of numerous parameters within and between dimensions. Both thermodynamic factors (e.g., stationary phase, mobile phase, temperature) and kinetic factors (e.g., gradient profile, sampling rate, column geometry, flow rates) strongly influence the analytical performance. To achieve effective method optimization, two‐dimensional liquid chromatography (2D‐LC) instrumentation‐related parameters must first be characterized, as they directly influence both method development and overall performance. In this work, we propose a practical guide (protocol) for comprehensive 2D‐LC system characterization. For clarity and ease of implementation, all calculations associated with this protocol can be performed using the *Characterization* module of the 2D‐LC Smart Calculator software, freely accessible online. The protocol usability was validated through inter‐laboratory testing. The impact of instrumental features on LC × LC performance was then assessed by in silico evaluation of the theoretical LC × LC performance of the characterized systems. The results clearly demonstrate the benefits of the proposed protocol, emphasizing the importance of system‐specific characterization for reliable LC × LC implementation. In addition, practical recommendations are provided to help users optimize their instruments when certain parameters are not adapted.

## 1 Introduction

Comprehensive two‐dimensional liquid chromatography (LC × LC) has gained increasing attention across application fields that require the analysis of complex samples, including pharmaceutical [1, 2, 3, 4, 5], environmental [6], food [7], and biological [8] analysis. By coupling two orthogonal separation mechanisms, LC × LC provides a resolving power that exceeds conventional one‐dimensional LC [9], thus enabling the resolution of analytical problems that were previously unattainable.

However, effective LC × LC method development remains challenging due to the strong interdependence of numerous thermodynamic and kinetic parameters that govern performance across both dimensions [10]. Although LC × LC optimization is inherently complex, several guidelines have been proposed to support the selection of appropriate operating conditions [11, 12, 13, 14]. Nevertheless, a critical and often overlooked step preceding method optimization, is the thorough characterization of the LC × LC system itself. Instrument‐related factors (such as system dispersion, dwell volume and system pressure) can substantially influence the achievable resolution and may limit the efficiency of the technique if not properly understood [9, 15]. To date, a practical guideline specific for two‐dimensional liquid chromatography (2D‐LC) system characterization has not yet been established.

In this work, we present a straightforward and broadly applicable protocol for 2D‐LC system characterization. The procedure is evaluated through inter‐laboratory testing to confirm its applicability across different instrument platforms. The LC × LC performance of the characterized systems is then examined in silico to illustrate how instrumental characteristics influence the separation capabilities of a comprehensive LC × LC method. These findings highlight the need for system‐specific assessment—and instrument reorganization when necessary—for the reliable and efficient implementation of LC × LC. In addition, practical guidance is provided to support users in reorganizing their systems when key instrumental parameters are not sufficiently optimized.

## 2 General Aspects

This protocol enables the determination of several system‐related parameters that influence 2D‐LC method optimization, performance, and data visualization. These parameters are briefly described below, and their detailed impact on chromatographic performance is further discussed in Section 4.

### 2.1 Extra‐Column Variance and Extra‐Column Volume

When an analyte is injected into an LC system, it migrates through the system and column as a discrete band. The observed peak width reflects dispersive processes occurring both within the column (column dispersion) and within the system components (extra‐column dispersion) [15]. The total observed peak variance (*σ* ^2^_tot_) can be expressed as:

(1) $$\sigma_{tot}^{2}=\sigma_{col}^{2}+\sigma_{ext}^{2}$$

where *σ* ^2^_col_ represents the column variance and *σ* ^2^_ext_ corresponds to the extra‐column variance.

*σ* ^2^_ext_ arises from the tubing and fittings comprised between the injector and the detector. To experimentally measure *σ* ^2^_ext_, the column is replaced by a zero‐dead‐volume union, thus eliminating *σ* ^2^_col_ from Equation (1) and directly allowing to measure *σ* ^2^_ext._

High *σ* ^2^_ext_ values decrease the separation performance and this effect is even more pronounced in LC × LC. Indeed, in typical LC × LC analyses, the ^2^D columns are short (commonly 3–5 cm), because on‐line coupling requires high flow rates to achieve sufficiently short ^2^D analysis times and thus maintain an adequate sampling rate for ^1^D peaks [15]. Furthermore, the column internal diameters are low (typically 2.1 mm) in order to take full advantage of the possibilities offered by ultra‐high performance liquid chromatography (UHPLC). Such small internal volumes in addition to very short gradient times in ^2^D drastically reduce *σ* ^2^_col,_ which makes the contribution of *σ* ^2^_ext_ (Equation 1) much more significant in ^2^D than in ^1^D where, on the other hand, gradient times are much longer. As a result, all sources of extra‐column dispersion must be minimized to preserve an acceptable balance between *σ* ^2^_ext_ and *σ* ^2^_col_.

Despite this requirement, commercial LC × LC instruments are often designed with flexibility and user convenience as priorities rather than minimization of extra‐column dispersion. Typical configurations (with separate pump, injector, thermostat, and detector modules connected by relatively long tubing) can therefore compromise performance under typical LC × LC conditions.

### 2.2 Dwell Volume

The dwell volume (*V_d_*) corresponds to the volume of the fluidic path between the point where the mobile phases are mixed in the LC pump and the column inlet [16]. To experimentally determine *V_d_*, mobile phase B is generally spiked with a tracer compound that serves as an indicator of solvent composition over time. After replacing the column with a zero‐dead‐volume union, a gradient from predominantly A to predominantly B is applied, and the signal of the tracer is monitored. As discussed later, the *V_d_* in ^1^D (^1^ *V_d_*) and ^2^D (^2^ *V_d_*) have a less pronounced impact on the chromatographic performance, compared to *σ* ^2^_ext_. However, *V_d_* can still affect the overall quality of LC × LC separations [16], especially in ^2^D as it reduces the available gradient time.

### 2.3 Time Offset

The time offset (*t*_offset_) corresponds to the delay between the start of the data acquisition and the actual injection valve switch. Although this parameter does not influence the practical performance of the method, it affects the visualization and alignment of chromatographic data. Because the time offset is instrument‐ and software‐dependent, its accurate determination is essential for correct data representation.

**FIGURE 1:**
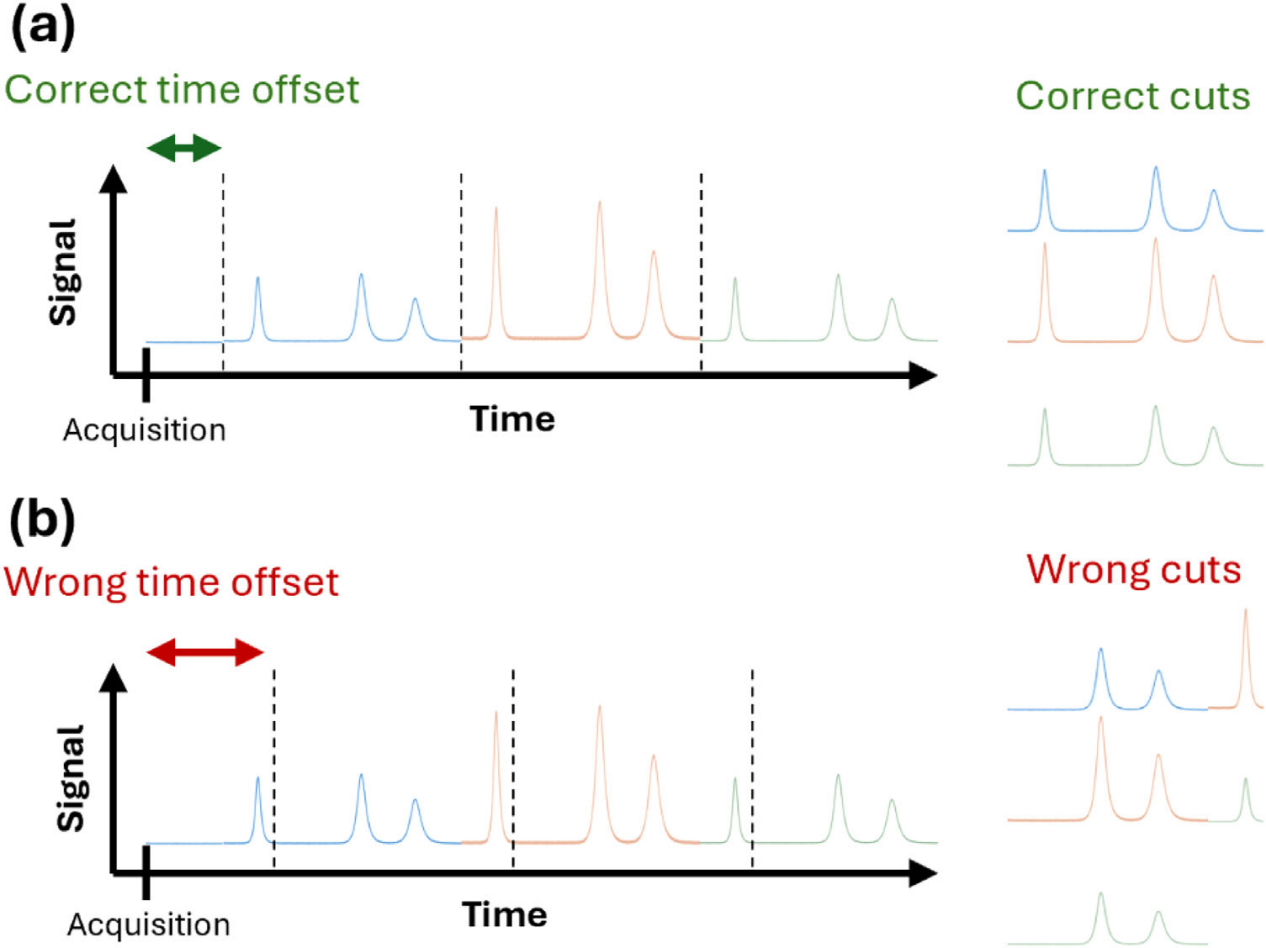
Effect of correct (a) and incorrect (b) determination of the second‐dimension time offset (i.e., the delay between 2D acquisition start and the actual 2D injection valve switching) on data alignment and cut reconstruction in LC × LC. The dashed vertical lines indicate the valve‐switching events.

In 2D‐LC systems, two detectors are typically used; therefore, the ^1^D time offset is defined as ^1^ *t*_offset_ and the ^2^D time offset as ^2^ *t*_offset._ The latter is particularly critical in full LC × LC, where modulation is typically performed using two identical sample loops. Fractions from ^1^D are continuously collected in these two loops, which are alternately filled and sequentially injected into ^2^D to ensure uninterrupted and comprehensive transfer of the ^1^D effluent. As a result, the ^2^D detector records a series of ^2^D chromatograms corresponding to each modulation cycle. These individual chromatograms must then be separated and recombined appropriately during data processing. As illustrated in Figure 1, an incorrect ^2^ *t*_offset_ characterization leads to a misalignment of the ^2^D chromatograms, which distorts the visualization and ultimately compromises the accuracy of the contour plot.

### 2.4 Extra‐Column Pressure

The extra‐column pressure (*P*_ext_) corresponds to the pressure generated by the instrument itself rather than the column. *P*_ext_ in the first dimension (^1^ *P*_ext_) is generally not critical, as flow rates are typically low. The main potential limitation is an insufficiently high system pressure to ensure stable operation at very low flow rates (5–20 µL/min). However, when using a ^1^D column, this situation is unlikely and, if it occurs, it can be readily mitigated by adding a restriction capillary. Consequently, ^1^ *P*_ext_ is not systematically monitored. In contrast, the *P*_ext_ in ^2^D (^2^P_ext_) is a critical parameter. Depending on its magnitude, the maximum achievable ^2^D flow rate—at a given pressure limit dictated by the column—may be constrained. Such limitations can directly impact the peak capacity and overall separation performance, as further increases in ^2^D flow rate may no longer be possible if ^2^ *P*_ext_ is too high.

## 3 Experimental

### 3.1 Chemicals and Reagents

Ultrapure water obtained from a Milli‐Q purification system (Millipore, Bedford, MA, USA) is used for the preparation of all mobile phases and aqueous solutions. LC–MS grade acetonitrile (ACN), and acetone (certified analytical reagent grade) are purchased from Thermo Fisher Scientific (Reinach, Switzerland). Methyl 4‐(hydroxymethyl)benzoate (98%) and lidocaine hydrochloride monohydrate are obtained from Sigma‐Aldrich (Buchs, Switzerland).

### 3.2 Samples and Mobile Phases

A mobile phase consisting of 40% ACN and 60% H_2_O (v/v) is prepared. For a total volume of 500 mL, 200 mL of ACN are combined with 300 mL of ultrapure water.

For dwell‐volume determination, mobile phase A consists of 250 mL of ultrapure water, while mobile phase B is prepared by adding 25 µL of acetone to 250 mL of ultrapure water to obtain a 0.1% (v/v) aqueous acetone solution.

Methylparaben and lidocaine stock solutions (1 mg/mL): A mass of 1.0 mg of methylparaben is accurately weighed into a 1.0 mL vial. Around 1 mL of the solvent mixture described in Section 3.2.1.1 (40% ACN and 60% H_2_O (v/v)) is then added, and the solution is vortexed until complete dissolution is achieved. This results in a methylparaben stock solution at a concentration of 1 mg/mL in 40:60 (v/v) ACN:H_2_O.

The same procedure is applied for lidocaine, resulting in a lidocaine stock solution at a concentration of 1 mg/mL in 40:60 (v/v) ACN:H_2_O.

Methylparaben working solution (0.01 mg/mL): Around 10.0 µL of the methylparaben 1 mg/mL stock solution is transferred into a 1.0 mL vial. A volume of 990 µL of the same 40:60 (v/v) ACN:H_2_O mixture is added, and the solution is vortexed. A final methylparaben concentration of 0.01 mg/mL in 40:60 (v/v) ACN‐H_2_O is thereby obtained.

Lidocaine working solution (0.001 mg/mL): 1.0 µL of the lidocaine 1 mg/mL stock solution is transferred into a 1.0 mL vial. A volume of 999 µL of the 40:60 (v/v) ACN:H_2_O mixture is then added, and the solution is vortexed. A final lidocaine concentration of 0.001 mg/mL in 40:60 (v/v) acetonitrile–water is thereby obtained.

### 3.3 Instrumentation and Software

The experiments are performed on a 1290 Infinity II series 2D‐LC instrument from Agilent Technologies (Waldbronn, Germany) including a ^1^D‐1290 binary pump, a ^2^D‐1290 high‐pressure binary pump (engineered to operate at high pressure), a 1290 thermostatted autosampler with a flow‐through needle injector equipped with a 20 µL sample loop, a ^1^D‐Multicolumn Thermostat (MCT) column compartment, a ^2^D‐Thermostatted Column Compartment (TCC), a ^1^D‐Variable Wavelength Detector (VWD), and a ^2^D‐diode‐array UV absorbance detector (DAD). The interface connecting the two dimensions is a 5‐position /10‐port Active Solvent Modulation (ASM) valve connected via four 1.9 µL transfer capillaries (170 × 0.12 mm) to two 2‐position/14‐port parking deck valves, mounted with two sample loops of 40 µL each. The pressure release kit (PRK) is installed to protect the ^1^D‐VWD flow cell from pressure spikes arising from the 2D‐LC valve switching. The second‐dimension separation is hyphenated either with a DAD operating at 80 Hz and a response time of 0.031 s, or with an Agilent 6530 Q‐TOF mass spectrometer equipped with a Dual Jet Stream ESI source. The MS is operated in positive ion mode with an extended dynamic range and an *m/z* range of 100–3200 (full scan). The following source parameters are applied: Gas temperature 325°C, gas flow 10 L/min, nebulizer pressure 20 psi, sheath gas temperature 400°C, and sheath gas flow 12 L/min. The capillary voltage (*V*_cap_) was set to 3500 V, nozzle voltage to 0 V, fragmentor to 200 V, skimmer to 65 V, and Oct 1 RF *V*_pp_ to 750 V. The instrument described here is referred as “*Agilent (1)*” in the manuscript. The other instruments used in this work and their specific modules used are described in Table S1.

Data acquisition was performed using Agilent MassHunter WorkStation Data Acquisition software. Data was processed with Agilent MassHunter Qualitative Analysis 12.0.

All calculations associated with this protocol can be done via the *Characterization* module of the 2D‐LC Smart Calculator software [17] developed by Heinisch et al.

The predicted peak capacities, sample dilution factors, and run numbers are obtained using the Optimization module of the 2D‐LC Smart Calculator software [17].

### 3.4 Instrument Configurations and Data Collection

#### 3.4.1 Determination of ^1^σ^2^_ext_, ^1^ *V*_ext_, and ^1^ *T*_offset_

**FIGURE 2:**
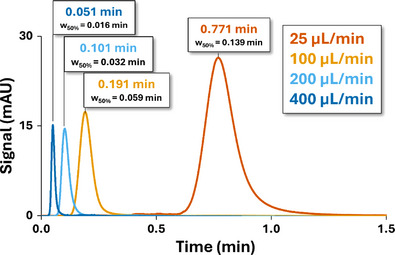
Retention time and w_50%_ measurements for methylparaben at four different flow rates (25 µL/min, orange; 100 µL/min, yellow; 200 µL/min, light blue; 400 µL/min, dark blue) in the first dimension of the LC × LC setup for ^1^σ^2^_ext_, ^1^V_ext_, and ^1^t_offset_ determination.

A solution of methylparaben at 0.01 mg/mL is used for the determination of ^1^ *σ* ^2^_ext_, ^1^ *V*_ext_, and ^1^ *t*_offset_. The instrument is configured to perform UV detection after the ^1^D column oven, and the detector outlet is directed to the waste to avoid connection to the ^2^D system as only ^1^D parameters are measured during this step. The conditions listed in Table 1 are then applied at a flow rate of 400 µL/min and repeated at flow rates of 200, 100, and 25 µL/min. A restriction capillary should preferably be used instead of the zero‐dead volume union to ensure sufficient backpressure for proper pump operation. Importantly, this restrictor must have a very low internal volume to minimize its contribution to ^1^ *σ* ^2^_ext_, ^1^ *V*_ext_. Therefore, a very short capillary with a narrow internal diameter should be used, typically around 25 µm. Retention times (*t_r_*) and peak widths at half height (*w*_50%_) of the methylparaben peaks are measured (as illustrated in Figure 2) and recorded for each flow rate (as reported in Table S2).

**TABLE 1:** Operating conditions for the ^1^D UV‐based measurement of ^1^D extra column variance, extra‐column volume and time‐offset evaluation.

Parameter	Condition
**Column**	Zero‐dead‐volume union
**Mobile phase**	40% ACN / 60% H_2_O (v/v)
**Flow rate**	400 µL/min (to be varied at 200, 100, and 25 µL/min)
**Injection volume**	0.5 µL
**Analyte**	Methylparaben, 0.01 mg/mL in 40% ACN / 60% H_2_O
**Detection**	UV 260 nm, acquisition rate = 80 Hz
**Column temperature**	Ambient
**Total analysis time**	5 min

#### 3.4.2 Determination of ^2^σ^2^_ext_, ^2^ *V*_ext_, ^2^ *P*_ext_, and ^2^ *T*_offset_

Either 0.01 mg/mL methylparaben (for UV detection in the ^2^D), or 0.001 mg/mL lidocaine (for MS detection in the ^2^D), is used. The system is configured as such that the effluent from the ^1^D directly enters the ^2^D immediately after the ^1^D column oven. When UV detection is used in ^1^D, the ^1^D UV detector is positioned between the column outlet and the ^2^D interface. The chromatographic conditions listed in Table 2 are then applied with a ^2^D flow rate of 1000 µL/min. This procedure is repeated at ^2^D flow rates of 1500, 2000, and 2500 µL/min. For each flow rate, the ^2^D extra‐column pressure (^2^ *P*_ext_), ^2^D *t_r_* and *w*_50%_ are measured for the most suitable peak (i.e., no peak saturation and correct signal to noise ratio). These measurements can be performed using either UV (as illustrated in Figure 4) or MS detection in ^2^D, depending on the configuration of the analytical setup.

**TABLE 2:** Operating conditions for the ^2^D measurement of ^2^σ^2^_ext_, ^2^V_ext_, ^2^P_ext_, ^2^time‐offset evaluation.

Parameter	Condition/Setting
**First dimension**	
Column	Zero‐dead‐volume union or short tubing that generates between 10 and 100 bar at 200 µL/min (e.g.: tubing with 25 cm length and 0.063 mm diameter)
Mobile phase	40% ACN / 60% H_2_O (v/v)
Flow rate	25 µL/min
Injection volume	0.5 µL of prepared standard solution
Analyte	Methylparaben (UV) or lidocaine (MS), in 40% ACN / 60% H_2_O
Detection	UV 80 Hz acquisition rate or no detection
Temperature	Ambient
**Interface/Modulation**	
Interface mode	Comprehensive
Sampling time	0.3 min
**Second dimension**	
Column	Zero‐dead‐volume union
Mobile phase	40% ACN / 60% H_2_O (v/v)
Flow rate	1000 µL/min (to be varied at 1500, 2000, and 2500 µL/min)
Detection (UV or MS)	UV: 260 nm, 80 Hz acquisition rate MS: >10 spectra/s, EIC = 235.1805 m/z (lidocaine)
Temperature	Ambient

#### 3.4.3 Determination of ^1^ *V_d_* and ^2^ *V_d_*

For ^1^ *V_d_* determination, a zero‐dead‐volume union is used in ^1^D. A UV detector is placed immediately after the union, and the detector outlet is directed to the waste to avoid any connection with the ^2^D system during this step. Mobile phases consisting of (A) 100% H_2_O and (B) 99.9% H_2_O / 0.1% acetone (v/v) are employed. The gradient shown in Figure S1 is applied at a flow rate of 200 µL/min (a higher flow rate can be used if the final plateau in the chromatogram is not clearly visible). Detection is performed at 254 nm.

For ^2^ *V_d_* determination, the ^1^D pumping system must remain operational; therefore, the ^1^D column is replaced with a zero‐dead‐volume union, and a low flow rate (0.1 mL/min of 40% ACN / 60% H_2_O) is employed in ^1^D. The instrument is configured for 2D‐LC operation with either ^2^D UV detection at 254 nm or ^2^D MS detection. The ^2^D column is replaced with a zero‐dead‐volume union. In the second dimension, a mobile phase consisting of (A) 100% H_2_O and (B) 99.9% H_2_O / 0.1% acetone is used; when MS detection is implemented, acetone needs to be replaced with an ionizable modifier (e.g. lidocaine). The gradient shown in Figure S1 is applied in the second dimension at a flow rate of 200 µL/min. The second dimension is flushed with the gradient without any injection. It is essential to ensure that the ^2^D gradient starts immediately and independently of any injection event. Depending on the 2D‐LC systems and software, this is achieved through different strategies, as listed in Table S4.

## 4 Results

### 4.1 Experimental Determination of the Relevant 2D‐LC System Parameters

#### 4.1.1 Determination of First Dimension Parameters

To determine *σ* ^2^_ext_, the peak width at half height (*w*_50%_) measurement was deliberately used instead of statistical moments. Indeed, implementing moment calculations would be less user‐friendly as it introduces additional manual data handling steps, thereby increasing the risk of operator‐dependent errors. As shown in Table S2, the w_50%_ values of methylparaben peaks at different ^1^D flow rates (*F*) allowed determination of ^1^ *σ* ^2^_ext_. Indeed, under the assumption of a gaussian peak, the variance can be determined at half the peak height by:

(2) $$\sigma_{ext}^{2}=\frac{{\left(w_{50\%}\times F\right)}^{2}}{5.54}\;$$

**FIGURE 3:**
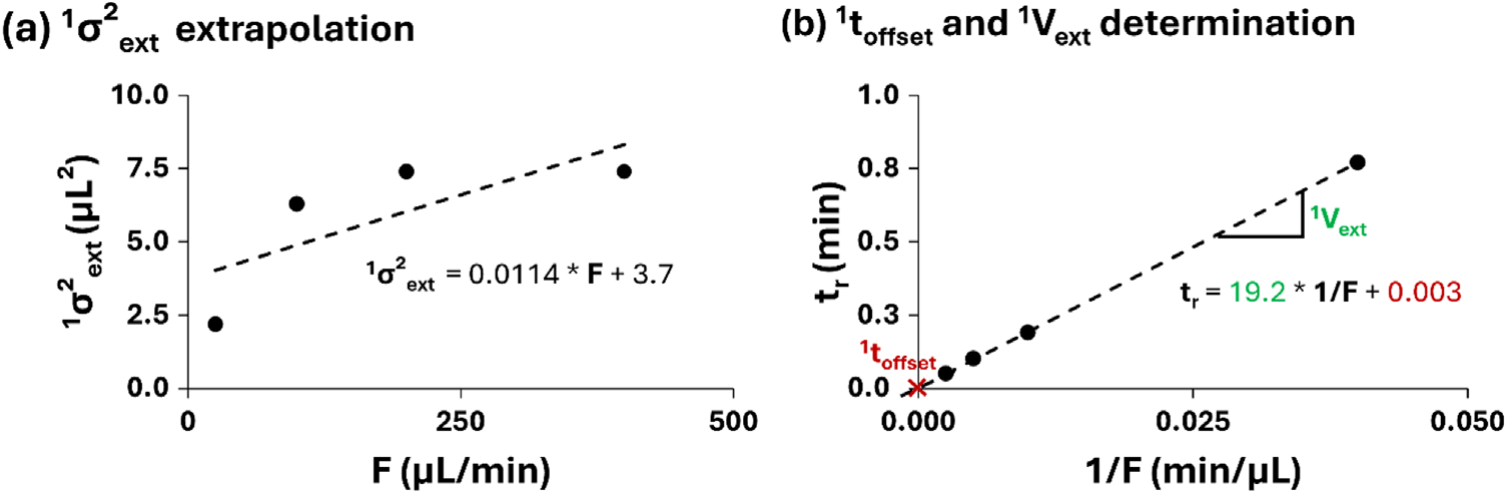
Plots of (a) ^1^σ^2^_e__x__t_ as a function of flow rate and (b) retention time as a function of 1/flow for methylparaben, with corresponding linear regressions. These plots were constructed using the data generated in Figure 2.

As illustrated in Figure 3a , ^1^ *σ* ^2^_ext_ values measured at different flow rates were used to establish a linear regression between ^1^ *σ* ^2^_ext_ and flow rate, allowing ^1^ *σ* ^2^_ext_ to be extrapolated to any desired first‐dimension flow rate relevant to a given application.

**FIGURE 4:**
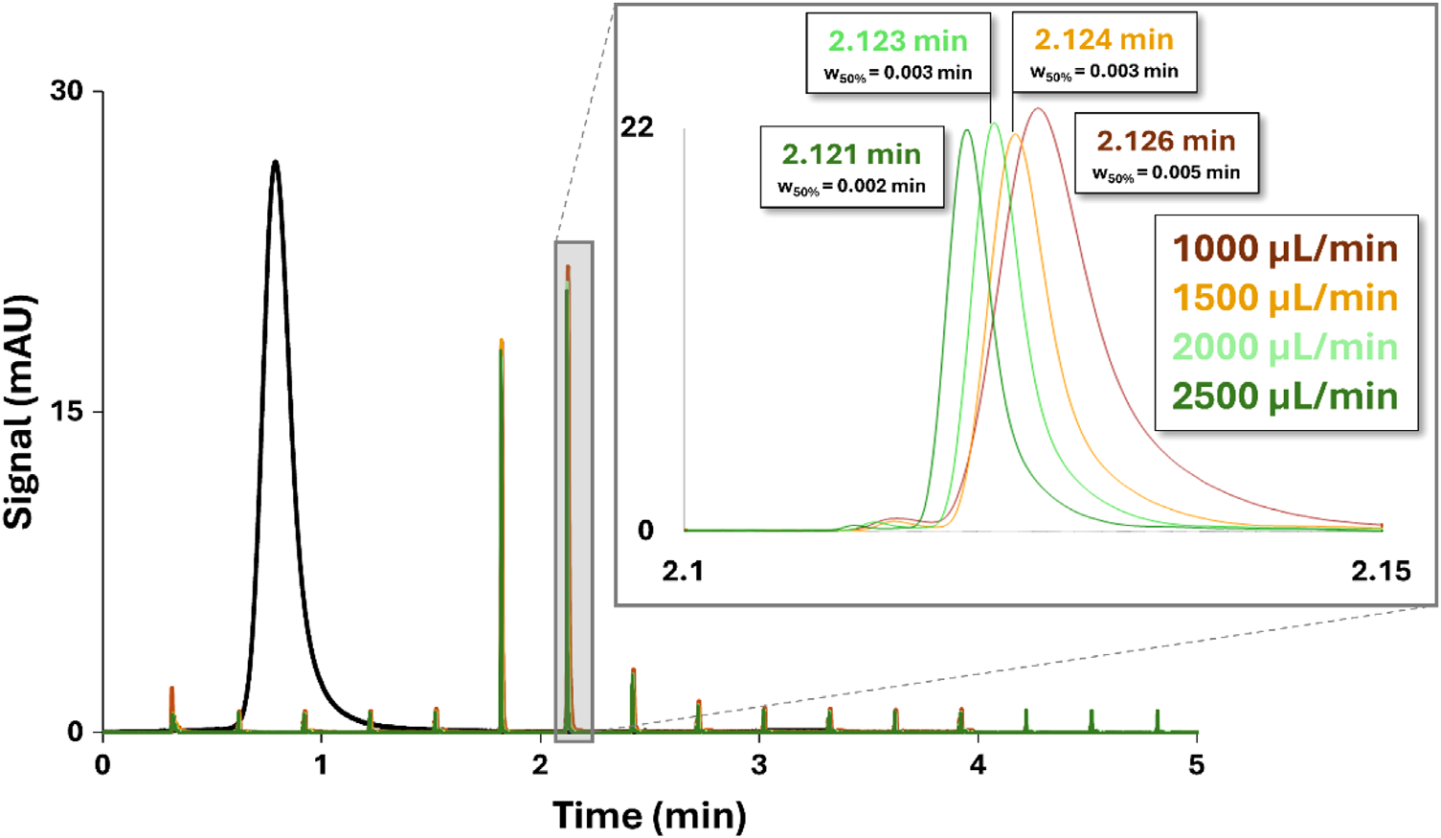
Retention time and w_50%_ measurements for methylparaben using UV detection at four different flow rates (1000 µL/min, brown; 1500 µL/min, yellow; 2000 µL/min, light green; 2500 µL/min, dark green) in the second dimension of the LC × LC setup for ^2^σ^2^_ext_, ^2^V_ext_, and ^2^t_offset_ determination. The black trace corresponds to the peak in the first dimension.

The retention times (*t_r_*) of the methylparaben peaks recorded at the various flow rates as shown in Table S2, are used to obtain the slope of the linear relationship between *t_r_* and the reciprocal *F* (1/*F*). As illustrated in Figure 3b, this slope directly provides the ^1^ *V*_ext_, while the intercept of the plot corresponds to the ^1^ *t*_offset_.

#### 4.1.2 Determination of Second Dimension Parameters

**FIGURE 5:**
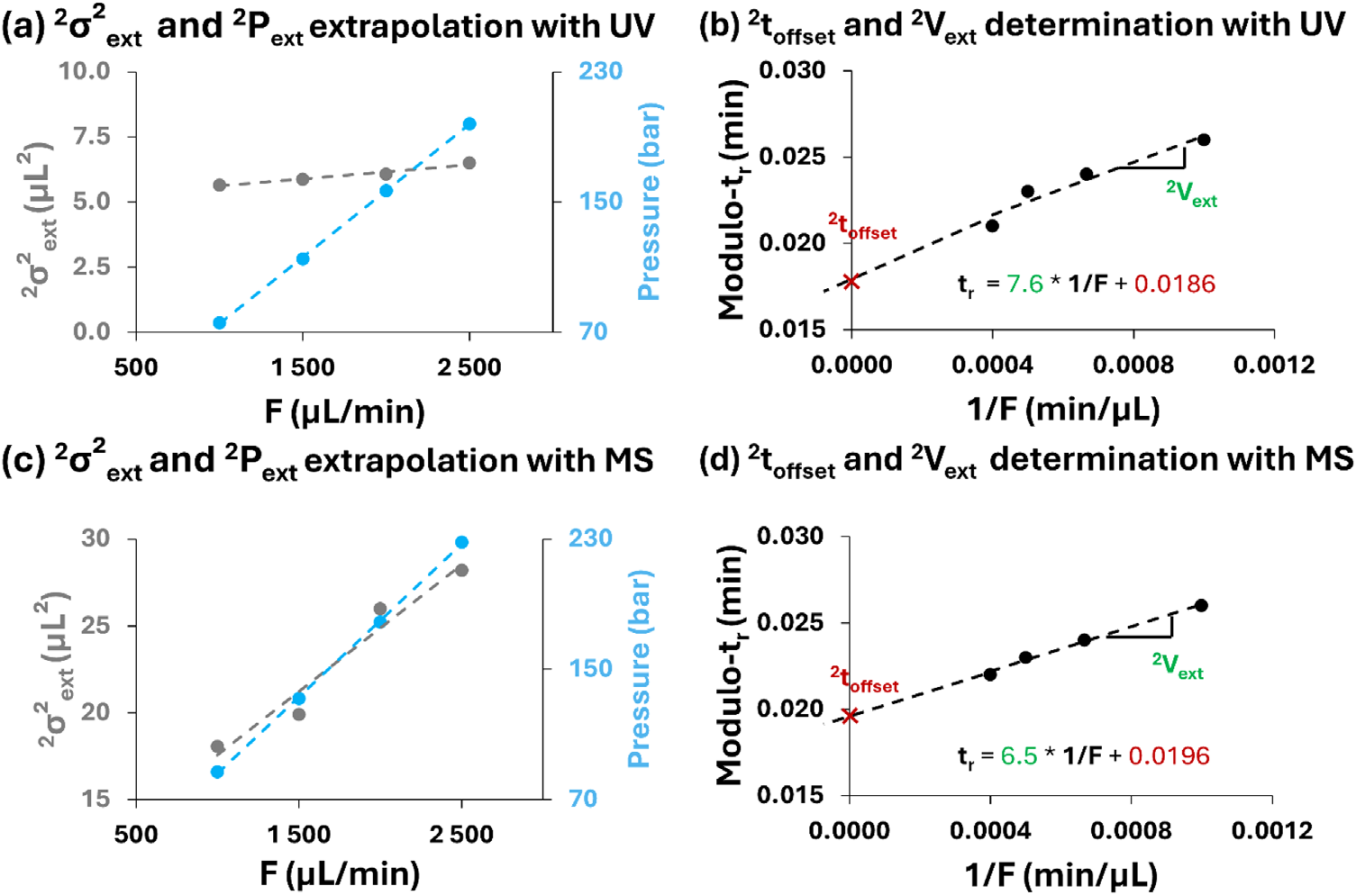
Plots of ^2^σ^2^_e__x__t_ (grey) and ^2^P_ext_ (blue) as a function of mobile phase flow rate for future extrapolation of parameters at any flow rate (a, c) and retention time as a function of 1/flow for ^2^t_offset_ and ^2^V_ext_ calculation (b, d) for methylparaben with UV detection (a, b) and lidocaine with MS detection (c, d). Linear regression fits are shown in all plots.

^2^ *P*_ext_ can also be monitored for all flow rates to plot ^2^ *P*_ext_ as a function of the ^2^D flow (blue trace in Figure 5a,c) to deduce the pressure that will be provided by the system when working at high flow rate for the LC × LC experiments.

The *w*_50%_ values of the methylparaben (for UV analysis) or lidocaine (for MS analysis) peak measured in ^2^D at different ^2^D flow rates allow determination of the evolution of ^2^ *σ* ^2^_ext_ as a function of flow rate (according to Equation 2) as shown in Table S3. The corresponding ^2^ *σ* ^2^_ext_ can then be established and extrapolated to any desired ^2^D flow rate as illustrated by the grey trace in Figure 5a,c.

The *t_r_* recorded at the different flow rates are first corrected using a modulo function based on the sampling time (0.3 min in this case; Table 2). Because the retention times measured for the compound detected in the second dimension at different flow rates do not necessarily correspond to the peak eluting during the first modulation cycle, a correction is required to reference all measurements to a given modulation event. Accordingly, each *t_r_* value is divided by the sampling time, and the remainder values of this division are then used to construct a linear relationship with the reciprocal of the flow rate (1/*F*), as shown in Figure 5b,d. The slope of this plot directly yields ^2^ *V*_ext_. The intercept of the plot corresponds to the ^2^ *t*_offset._

#### 4.1.3 Determination of Dwell Volumes

^1^ *V_d_* is determined based on the ^1^D experiments described in Section 3.5.3 and ^2^ *V_d_* is determined based on the ^2^D experiments described in Section 3.5.4. The *t*_50_ values in ^1^D and in ^2^D are determined from the initial and final plateau of the gradient performed in ^1^D and in ^2^D (to calculate ^1^ *V_d_* and ^2^ *V_d_* respectively), according to the procedure illustrated in Figure S1. Based on the measured value of *t*_50_, the dwell time (*t_d_*) can be obtained using the following equation:

(3) $$t_{d}=t_{50}\;-\frac{1}{2}t_{g}-2\;\min$$

The corresponding dwell volume (*V_d_*) is then calculated from the flow rate using:

(4) $$V_{d}=\left(t_{50}-\frac{1}{2}t_{g}-2\;\min\right)\cdot F$$

### 4.2 Validation of the Automated Protocol Across Multiple Laboratories

To improve ease of use and ensure high‐quality guidance, all protocol steps, experimental procedures, and data processing described above can be supported by the *Characterization* module of the 2D‐LC Smart Calculator software [17].

The usability of this protocol was evaluated through inter‐laboratory testing. The study included four Agilent 1290 Infinity 2D‐LC systems and one Shimadzu Nexera‐e system. Instrument modules and configurations are summarized in Table S1, and tubing dimensions are provided in Table 3. The 2D‐LC system‐characterization procedure—supported by the *Characterization* module of the 2D‐LC Smart Calculator software [17]—was successfully applied by all participating laboratories, regardless of the instrument model or configuration, demonstrating that the protocol is broadly applicable. The measured 2D‐LC systems parameters are reported in Table 4 for each instrument.

**TABLE 3:** Main characteristics and tubing configurations of the different 2D‐LC systems used in this work (four different Agilent 1290 Infinity II 2D‐LC Solution and one Shimadzu Nexera‐e).

System	^1^D mixer volume	^2^D mixer volume	Interface	^1^D tube column to UV	^2^D tube column to detector	^1^D UV flow cell volume	^2^D UV flow cell volume
**Agilent (1)**	35 µL	35 µL	5‐position /10‐port ASM valve (G4243A) connected via four 1.9 µL transfer capillaries (170 × 0.12 mm) to two 2‐position/14‐port parking deck valves (MHC), mounted with two sample loops of 40 µL each (420 × 0.35 mm)	28 cm × 100 µm	34 cm × 100 µm	2 µL	1 µL
**Agilent (2)**	35 µL	35 µL	2‐position /4‐port duo valve (5067‐4244) with two sample loops of 40 µL each (815 × 0.25 mm)	15 cm × 127 µm	15 cm × 100 µm (+ divert valve when MS was used)	1 µL	1 µL
**Agilent (3)**	35 µL	35 µL	2‐position /4‐port duo valve (5067‐4244) with two sample loops of 20 µL each (637 × 0.2 mm)	25 cm × 100 µm	32 cm × 100 µm	13 µL	1 µL
**Agilent (4)**	No external mixer	35 µL	5‐position/10 port ASM valve (G4243A) with two sample loops of 60 µL each (831 × 0.35 mm)	20 cm × 127 µm	15 cm × 127 µm	2 µL	1 µL
**Shimadzu (1)**	20 µL	20 µL	Two high speed/high pressure 2‐position, 6‐port valves (FCV‐0206H3) with microelectric actuator, with two sample loops of 10 µL each (210 × 0.25 mm)	55 cm × 100 µm	55 cm × 100 µm	8 µL	8 µL

**TABLE 4:** Characterization of various 2D‐LC instruments (four different Agilent 1290 Infinity II 2D‐LC Solution and one Shimadzu Nexera‐e). All the values were experimentally obtained using the protocol previously described to characterize 2D‐LC instruments. ^1^D values for 0.2 mL/min and ^2^D values for 2.6 mL/min.

	Time offset			Dwell volume		Extra column variance			Extra column volume			Extra column pressure	
System	^1^t_offset_ (s) (UV)	^2^t_offset_ (s) (UV)	^2^t_offset_ (s) (MS)	^1^V_D_ (µL)	^2^V_D_ (µL)	^1^σ^2^_ext_ [µL^2^] (UV)	^2^σ^2^_ext_ [µL^2^] (UV)	^2^σ^2^_ext_ [µL^2^] (MS)	^1^V_ext_ [µL] (UV)	^2^V_ext_ [µL] (UV)	^2^V_ext_ [µL] (MS)	^2^P_ext_ (bar) (UV)	^2^P_ext_ (bar) (MS)
**Agilent (1)**	0.18	1.11	1.18	136	86	6	5	29	19	8	6	212	244
**Agilent (2)**	0.30	2.30	2.30	180	90	5	7	134	15	14	17	122	112
**Agilent (3)**	0.54	0.31	—	808	122	15	12	—	29	17	—	138	—
**Agilent (4)**	−2.34	0.64	—	838	92	6	33	—	25	15	—	227	—
**Shimadzu (1)**	−0.84	1.06	1.16	212	94	143	53	406	63	9	49	94	45

To illustrate the impact of those parameters on global performance, the *Optimization* module of the 2D‐LC Smart Calculator [17] software was used. First, the values obtained with the system *Agilent 1* and UV detection (see values in Table 4) were used in the Calculator to simultaneously optimize three parameters: (i) Peak capacity, which reflects the separation power and should be maximized; (ii) dilution factor, which represents sample dilution induced by the 2D process and should be minimized to ensure acceptable sensitivity; and (iii) the number of second‐dimension runs, which is directly related to total analysis time and is ideally reduced. This in silico optimization enables the determination of optimal values for 1D‐column length, sampling time and 1D‐flow‐rate. Those in silico optimized conditions for this 2D‐LC analysis are listed in Figure S2 and summarized in Table S5. Once the conditions were optimized for the *Agilent* (*1*) instrument, the parameters of the 2D‐LC system were studied independently of each other with values ranging from the lowest to the highest, in accordance with the parameter values obtained with the different instruments (Table 4).

**FIGURE 6:**
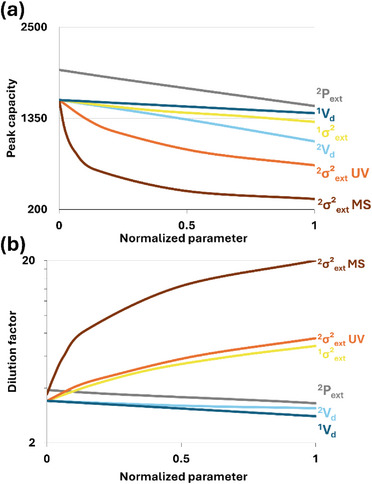
Impact of different parameters ^2^P_ext_ (grey trace), ^1^V_d_ (dark blue trace), ^2^V_d_ (light blue trace), ^1^σ^2^_e__x__t_ (yellow trace), ^2^σ^2^_e__x__t_ with UV detection (orange trace) and ^2^σ^2^_e__x__t_ with MS detection (brown trace) on (a) the effective peak capacity and (b) dilution factor in an LC × LC setup under representative operating conditions (Table S5). All the parameters are normalized for graphical representation.

The influence of these parameters on the instrument performance is illustrated in Figure 6. As the run number varies only with the total analysis time and therefore changes in ^1^ *V_d_*, and this to a very limited extent, the present result analysis focuses solely on the peak capacity and the dilution factor, which are used as performance indicators (Figure 6).

### 4.3 Interlaboratory Comparison and Discussion

#### 4.3.1 Time Offset

As shown in Table 4, time offset values need to be characterized as they are highly instrument‐ and software‐dependent. Depending on the situation, the time offset can be positive (the sample is injected *after* the acquisition starts) or negative (the sample is injected *before* the acquisition starts). Although these values are important for data visualization and alignment (as illustrated in Figure 1), they do not affect chromatographic performance.

#### 4.3.2 System Pressure

As shown in Table 4, ^2^ *P*_ext_ values are instrument‐dependent and can vary between 45 to 244 bar (for a fixed ^2^D flow rate of 2600 µL /min), depending on the instrument and especially the tubing geometry (length and internal diameter). To assess the specific impact of ^2^ *P*_ext,_ a ^2^D upper pressure limit of 1100 bar was considered, and the highest flow rate that can be used at this pressure limit was considered to calculate the corresponding peak capacity, and dilution factor, thereby highlighting the effect of ^2^ *P*_ext_ on performance (Figure 6, grey trace). Indeed, as ^2^ *P*_ext_ directly limits the maximum flow rate that can be applied, the allowable ^2^D flow rate varies between instruments. Because peak capacity depends on flow rate, high ^2^ *P*_ext_ values restrict the attainable flow rate and consequently reduce peak capacity. This is illustrated in Figure 6a (grey trace), showing a decrease in peak capacity when instruments with high ^2^ *P*_ext_ are used, whereas impact on dilution factor is negligible (Figure 6b, grey trace). As ^2^D separations typically operate at the highest possible flow rates, it is essential to anticipate the pressure generated at any given flow rate to avoid exceeding the system's pressure capability. The tube diameter is the key factor for external pressure, but it also has a significant impact on solute dispersion. It is therefore essential to find the right balance between external pressure and extra‐column variance, as discussed later.

#### 4.3.3 Dwell Volumes

The ^1^ *V_d_* varies substantially between instruments from 136 to 838 µL. With modern binary ^1^D pumps such as the Agilent 1290 or the Shimadzu Nexera LC‐40B X3, ^1^ *V_d_* values are typically around 200 µL, or even lower when equipped with the smallest available mixers. Larger ^1^ *V_d_* may be encountered with quaternary pump designs, such as the Agilent 1260 series. However, even if the dwell volume can impact retention and selectivity [16], the main impact of a high ^1^ *V_d_* is a substantial increase in analysis time. Indeed, the main practical consequence of a large ^1^ *V_d_* is due to the low ^1^D flow rates typically used in LC × LC: A large ^1^ *V_d_* increases the initial delay of the ^1^D separation, resulting in unnecessary loss of analysis time and increase in run number. However, the impact of ^1^ *V_d_* on overall 2D‐LC performance remains limited. As illustrated in Figure 6a,b (dark blue trace), neither the effective peak capacity nor the dilution factor is significantly affected by changes in ^1^ *V_d_*.

In contrast, a large ^2^ *V_d_* has a significant impact on effective peak capacity. To reduce ^2^ *V_d_*, a strategy is to select the smallest available mixer (typically 20 or 35 µL depending on the provider) and reduce the internal diameter of the tubing between the pumps and the injector. However, as shown in Table 4, all instruments exhibit a ^2^ *V_d_* of approximately 90 µL (excluding the injection loop). The main difference therefore arises from the loop volume, which must be adapted to the injected volume. Indeed, when ^2^ *V_d_* is increased in silico to mimic loop volumes of 50, 100, and 150 µL, a non‐negligible loss in peak capacity is observed, reaching up to −33% (Figure 6a, light blue trace). This can be explained by the systematic delay induced by the increase of ^2^ *V_d_* at the beginning of each ^2^D gradient. This reduces the effective gradient time available within each modulation cycle and therefore the overall peak capacity while having a very slight impact on the dilution factor (Figure 6b, light blue trace).

#### 4.3.4 Extra Column Variances

Typical Agilent 1200 systems exhibit extra‐column variances in the range of 10–20 µL^2^ [15]. As shown in Table 4, this trend is confirmed for most of the Agilent instruments with UV detection. Higher values are explained by tubing with a 127 µm internal diameter instead of 100 µm tubing downstream of the 2D‐LC column oven. Furthermore, similar *σ* ^2^_ext_ values were obtained in D^1^ at 200 µL/min and in D^2^ at 2600 µL/min. Although this may appear unexpected, it can be explained by the highly optimized configuration in D^2^, which features very low volumes compared with the configuration used in D^1^. In addition, both ^1^ *σ* ^2^_ext_ and ^2^ *σ* ^2^_ext_ are consistently higher for the Shimadzu instrument compared with the Agilent systems. This difference may arise from the intrinsically larger internal volumes of the Shimadzu platform, from the longer lengths of tubing required to interconnect the modules, or from the higher‐volume UV flow cell used for this system.

The influence of *V*_ext_ is typically negligible for large‐volume columns—where *σ* ^2^_col_ dominates *σ* ^2^_tot_—or under low flow rate conditions [15]. Consequently, ^1^D, in a 2D‐LC system, is usually less affected by extra‐column effects than ^2^D (as shown in Figure 6, yellow trace). For this reason, low importance is usually given to reduce ^1^ *σ* ^2^_ext_ (as illustrated in Table 4 with ^1^ *σ* ^2^_ext_ values ranging from 5 to 143 µL^2^). However, as illustrated in Figure 6b (yellow trace), an increase in *σ* ^2^ext leads to a higher dilution factor, since broader peaks in the first‐dimension result in greater dilution than narrower ones.

Nevertheless, the impact of ^2^ *σ* ^2^_ext_ is more important; ^2^D employs small‐volume columns with steep gradients, resulting in peaks that are intrinsically narrow and highly sensitive to dispersion [15, 18]. As illustrated in Table 4, the ^2^ *σ* ^2^_ext_ is highly dependent on the instrumentation. However, as shown in Figure 6 (orange trace), increasing ^2^ *σ* ^2^_ext_ from 5 to 50 µL^2^ reduces the peak capacity and increases the dilution factor by a factor greater than two, making ^2^ *σ* ^2^_ext_ the most critical factor in LC × LC.

To reduce ^1^ *σ* ^2^_ext_ and ^2^ *σ* ^2^_ext_, several technical solutions can be implemented. The easiest strategy is to use tubing with the shortest possible length and, above all, the smallest internal diameter—particularly downstream of the second‐dimension column—since extra‐column dispersion scales with the fourth power of the internal diameter [18]. This approach is strongly recommended, even after installation of the 2D‐LC system by the instrument provider, as factory installations are not always optimal (e.g., module placement or tubing selection). This approach may, however, involve compromises, as excessively narrow tubing can lead to an undesired increase in pressure. The use of tubing and fittings from the same manufacturer as the instrument is also advised to avoid mismatched connections that introduce additional dead volume.

The contribution of the detector to ^1^ *σ* ^2^_ext_ and ^2^ *σ* ^2^_ext_ can be lowered by working with a detector flow cell with lower volumes (1 µL or less), although this may potentially impact the background noise and limits of detection. Furthermore, MS detection should be used with high cautions, as the MS interface can introduce substantial additional volumes (connection tubing, nebulizer probe tubing) that significantly increases ^2^ *σ* ^2^_ext_ [19, 20, 21], as illustrated in Figure 6. If MS‐compatible mobile phases are used in both dimensions, the use of a divert valve prior to the ^2^D detector should be avoided, as it greatly increases ^2^ *V*_ext_ (c.f. Table 4).

Coiled loops and coiled tubing are preferred over straight configurations for the sample loops, as straight loops represent the worst‐case scenario in terms of band broadening during loop filling and emptying [22]. Furthermore, a “First‐In–Last‐Out” (countercurrent) filling loop strategy is recommended for second‐dimension injections, since greater dispersion occurs when the loop is filled in the same direction in which it is emptied [23].

**FIGURE 7:**
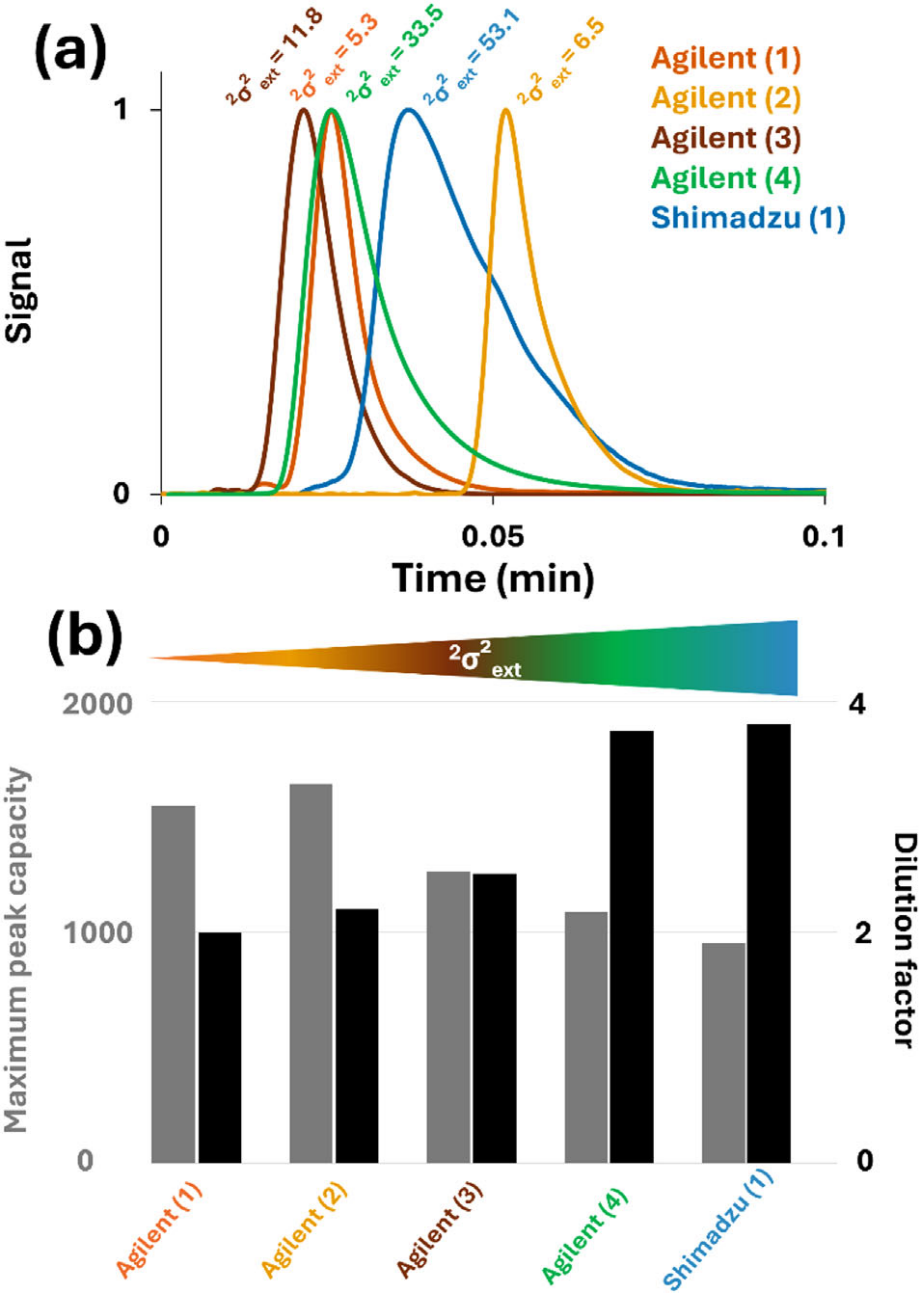
(a) Superimposed ^2^D UV chromatograms used for the determination of ^2^σ^2^_ext_ for the five instruments included in the study. The methylparaben peak intensity was normalized across all datasets, and retention times were corrected using a modulo function to align all signals within the same modulation cycle. (b) Optimized effective peak capacities (grey) and dilution factor (black) obtained after instrument‐specific in silico optimization for each specific instrument.

The importance of system characterization and instrumental optimization prior to 2D‐LC analysis is further illustrated in Figure 7. Indeed, one might assume that comparable performance could still be achieved across instruments, even if they are not optimally configured in practice, provided that the method conditions are specifically optimized for each system. To evaluate this hypothesis, an instrument‐specific in silico optimization was performed using the *Optimization* module of the 2D‐LC Calculator. Starting from the initial conditions reported in Table S5, the highest flow rates compatible with a maximum overall second‐dimension pressure of 1100 bar were applied for each instrument. Instrument‐specific experimentally determined values of *P*_ext_, *σ* ^2^_ext_, and *V_d_* (with UV detection) were incorporated into the simulations. As illustrated in Figure 7, the overall performance of the instrument is clearly governed by ^2^ *σ* ^2^_ext._ After simulation of the maximum effective peak capacities and the corresponding dilution factors obtained via Pareto optimization for each 2D‐LC instrument (Figure 7b), the conclusion is straightforward: Instruments exhibiting high ^2^ *σ* ^2^_ext_ values (reflected by larger peak widths at 50% height in Figure 7a) show a markedly reduced maximum peak capacity and a significantly increased dilution factor compared to systems with low ^2^ *σ* ^2^_ext_, even after instrument‐specific optimization. These results highlight the importance of this protocol, as thorough instrument characterization is essential to ensure practical optimization prior to method development. Even under optimal operating conditions, LC × LC performance remains strongly constrained by the instrumental setup itself.

## 5 Conclusions

This work provides a practical and broadly applicable protocol for 2D‐LC system characterization, which can be supported either by the *Characterization* module of the Excel tool *2D‐LC Smart Calculator*, which can be downloaded for free [17], or carried out independently by following the procedure detailed in this article.

Through inter‐laboratory evaluation across multiple platforms and configurations, the usability of the described procedure was confirmed, and the collected data demonstrated that instrument‐related parameters (particularly extra‐column variance, dwell volumes, and second‐dimension pressure limitations) vary substantially between systems and can markedly influence achievable dilution factors, peak capacities, run number, and overall separation performance in LC × LC.

Importantly, this characterization is intended to be performed once for a given instrument configuration and does not need to be repeated for each new LC × LC method, unless modifications to the system configuration are made. Moreover, the protocol relies on inexpensive and readily available compounds (e.g., lidocaine, methylparaben, and acetone) and can be completed within approximately two days for a full system characterization. By enabling users to quantify these parameters and anticipate their impact on method performance, the protocol provides a solid foundation for informed method development and instrument optimization. Adoption of this characterization procedure and application of the practical recommendations to optimize instruments will facilitate the development of comprehensive LC × LC methods, thereby supporting a wider and more effective use of 2D‐LC in complex analytical applications.

## Author Contributions

**Megane K. Aebischer**: writing – original draft, investigation, visualization, conceptualization. **Marie Pardon**: writing – review and editing, investigation. **Clémence Gadot**: writing – review and editing, investigation. **Katia Arena**: writing – review and editing, investigation. **Niklas Carstensen**: writing – review and editing, investigation. **Michael Laemmerhofer**: writing – review and editing. **Paola Dugo**: writing – review and editing. **Francesco Cacciola**: writing – review and editing. **Luigi Mondello**: writing – review and editing. **Morgan Sarrut**: writing – review and editing. **Deirdre Cabooter**: writing – review and editing. **Sabine Heinisch**: writing – original draft, conceptualization. **Davy Guillarme**: writing – original draft, supervision, conceptualization.

## Conflicts of Interest

The authors declare no conflicts of interest.

## Supporting information




**Supporting File**: jssc70434‐sup‐0001‐SuppMat.docx.

## Data Availability

The free and open‐access 2D‐LC Smart Calculator (Excel‐based tool) is available at: https://farma‐unites.unige.ch/en/guillarme‐lab/tools/2d‐lc‐smart‐calculator
