# Supplementary material for: A Protocol for Characterizing Comprehensive Two‐Dimensional Liquid Chromatography Systems
Source: J Sep Sci. 2026 May 11;49:e70434. doi: 10.1002/jssc.70434 (PMC13159402; doi:10.1002/jssc.70434)

**A protocol for characterizing comprehensive two-dimensional liquid chromatography systems**

**Supplementary materials**

Megane K. AEBISCHER^1,2^, Marie PARDON^3,4^, Clémence GADOT^5^, Katia ARENA^6^, Niklas CARSTENSEN^8^, Michael LAEMMERHOFER^8^, Paola DUGO^6,7^, Francesco CACCIOLA^6^, Luigi MONDELLO^6,7^, Morgan SARRUT^5^, Deirdre CABOOTER^3^, Sabine HEINISCH^9^, Davy GUILLARME^1,2^

^1^ School of Pharmaceutical Sciences, University of Geneva, CMU - Rue Michel Servet 1, 1211 Geneva 4, Switzerland

^2^ Institute of Pharmaceutical Sciences of Western Switzerland, University of Geneva, CMU - Rue Michel Servet 1, 1211 Geneva 4, Switzerland

^3^ Laboratory for Pharmaceutical Analysis, Department of Pharmaceutical and Pharmacological Sciences, KU Leuven, Herestraat 49 box 923, 3000, Leuven, Belgium

^4^ Laboratory for Molecular Biodiscovery, Department of Pharmaceutical and Pharmacological Sciences, KU Leuven, Herestraat 49 box 824, 3000, Leuven, Belgium

^5^Syensqo Lyon Research and Innovation Center, 85 avenue des Frères Perret,
69190 Saint-Fons, France

^6^ Messina Institute of Technology c/o Department of Chemical, Biological, Pharmaceutical and Environmental Sciences, University of Messina, Viale G. Palatucci 13, 98168 – Messina, Italy

^7^Chromaleont s.r.l., c/o Department of Chemical, Biological, Pharmaceutical and Environmental Sciences, University of Messina, Viale G. Palatucci 13, 98168 – Messina, Italy

^8^ Institute of Pharmaceutical Sciences, Pharmaceutical (Bio-)Analysis, University of Tübingen, Auf der Morgenstelle 8, 72076 Tübingen, Germany.

^9^ Université de Lyon, Institut des Sciences Analytiques, UMR 5280 CNRS, 5 rue de la Doua, 69100 Villeurbanne, France

**Correspondence:** Prof. Davy Guillarme, Institute of Pharmaceutical Sciences of Western Switzerland (ISPSO), University of Geneva, Group of Analytical Pharmaceutical Chemistry
CMU - Rue Michel Servet, 1, 1206 Geneva – Switzerland; Phone: +41.22.379.34.63; Email: davy.guillarme@unige.ch

**Keywords:** 2D-LC, bidimensional liquid chromatography, comprehensive, instrumentation, dispersion, characterization, dwell volume

**Table S1.** Specific modules of the five instruments used for the inter-laboratory protocol validation.

| **System** | **^1^D pump** | **^1^D sampler** | **^1^D UV detector** | **Interface** | **^2^D pump** | **^2^D UV detector** | **^2^D MS detector** |
| --- | --- | --- | --- | --- | --- | --- | --- |
| **Agilent (1)** | 1290 Infinity Binary Pump (G4220A) | 1290 Infinity AutoSampler (G4226A) | 1260 Variable Wavelength Detector (G7114A) | 5-position /10-port ASM valve (G4243A)  connected via four 1.9 µL transfer capillaries (170 × 0.12 mm) to two 2-position/14-port parking deck valves (MHC), mounted with two sample loops of 40 µL each (420 × 0.35 mm) | 1290 Infinity High Speed Pump (G7120A) | 1260 Infinity Diode Array Detector (G4212B) | 6530 Q-TOF (G6530C) |
| **Agilent (2)** | 1290 Infinity High Speed Pump (G7120A) | 1290 Infinity Vialsampler (G7129B) | 1290 Infinity Diode Array Detector (G7117A) | 2-position /4-port duo valve  (5067-4244)  with two sample loops of 40 µL each (815 × 0.25 mm) | 1290 Infinity High Speed Pump (G7120A) | 1290 Infinity Diode Array Detector (G7117A) | 6530 Q-TOF (G6530C) |
| **Agilent (3)** | 1260 Infinity Binary Pump (G7112B) | 1290 Infinity Vialsampler (G7129A) | 1260 Infinity Diode Array Detector (G7115A) | 2-position /4-port duo valve  (5067-4244)  with two sample loops of 20 µL each (637 × 0.2 mm) | 1290 Infinity High Speed Pump (G7120A) | 1290 Infinity Diode Array Detector (G7117A) | - |
| **Agilent (4)** | 1260 Infinity II Bioinert Quaternary Pump (G5654A) | 1290 Infinity AutoSampler (G4226A) | 1290 Variable Wavelength Detector (G7114B) | 5-position/10 port ASM valve  (G4243A)  with two sample loops of 60 µL each (831 × 0.35 mm) | 1290 Infinity Binary Pump (G4220A) | 1290 Infinity Diode Array Detector (G4212A) | - |
| **Shimadzu (1)** | Nexera Binary pump (LC-40B X3) | Nexera AutoSampler (SIL-40C X3) | Nexera Photodiode Array Detector (SPD-M40 PDA Detector) | Two high speed/high pressure 2-position, 6-port valves  (FCV-0206H3)  with microelectric actuator, with two sample loops of 10 µL each (210 × 0.25 mm) | Nexera Binary pump  (LC-40B X3) | Nexera Photodiode Array Detector (SPD-M40 PDA Detector) | TQ LCMS-8050 |

**Table S2.** Measured retention times, peak-widths, and calculated ^1^σ²ₑₓₜ values for methylparaben peaks at various flow rates in ^1^D.

| **Flow (µL/min)** | **1/Flow (min/µL)** | **Measured retention time (min)** | **Measured half peak width (min)** | **Calculated ^1^σ²_ext_ (µL²)** |
| --- | --- | --- | --- | --- |
| 25 | 0.040 | 0.771 | 0.139 | 2.2 |
| 100 | 0.010 | 0.191 | 0.059 | 6.3 |
| 200 | 0.005 | 0.101 | 0.032 | 7.4 |
| 400 | 0.003 | 0.051 | 0.016 | 7.4 |

**Table S3.** Measured retention times, peak-widths, extra column pressure and calculated ^2^σ²ₑₓₜ values for methylparaben (with UV detection) or lidocaine (with MS detection) peaks at various flow rates.

| **Flow-rate (µL/min)** | **1/Flow (min/µL)** | **Measured retention time (min)** | | **Modulo-corrected retention times (min)** | | **Measured half peak width (min)** | | **Calculated ^2^σ²_ext_ (µL²)** | | **Measured extra column pressure (bar)** | |  |
| --- | --- | --- | --- | --- | --- | --- | --- | --- | --- | --- | --- | --- |
|  |  | UV | MS | UV | MS | UV | MS | UV | MS | UV | MS | |
| 1000 | 0.0010 | 2.126 | 3.026 | 0.026 | 0.026 | 0.005 | 0.010 | 4.5 | 18.1 | 76 | 87 | |
| 1500 | 0.0007 | 2.124 | 3.024 | 0.024 | 0.024 | 0.003 | 0.007 | 3.7 | 19.9 | 115 | 132 | |
| 2000 | 0.0005 | 2.123 | 3.023 | 0.023 | 0.023 | 0.003 | 0.006 | 6.5 | 26.0 | 157 | 179 | |
| 2500 | 0.0004 | 2.121 | 3.022 | 0.021 | 0.022 | 0.002 | 0.005 | 4.5 | 28.2 | 198 | 228 | |

**Table S4.** Strategies to measure ^2^V_d_ ensuring the ^2^D gradient starts immediately and independently of any injection event.

| **Strategy** | **^2^V_d_ measurement description** |
| --- | --- |
| **Strategy 1** | \|  \| \| --- \|  \| **A flush gradient is configured at the beginning of the method** by selecting the “*Copy from Analytical Gradient*” option before initiating the 2D gradient. \| \| --- \| |
| **Strategy 2** | In the instrument configuration, 2D-LC mode is not enabled. This allows the user to create a **standalone method for the ^2^D pump in the regular method editor** (not in the 2D method editor) and to run it without any injection or ^1^D flow. |
| **Strategy 3**  *(not recommended)* | When a heart-cut or a comprehensive run must be performed to trigger data acquisition in the second dimension, **the gradient can be applied manually**. After a 2-min wait, %B is instantaneously changed from 1% to 99% in the control software, and the moment at which the pressure shock is detected (no chromatographic signal recorded) is used for timing. |

**Table S5.** *In silico* conditions used in the *Optimization* module of the 2D-LC Smart Calculator for the evaluation of the impact of **^1^**σ²_ext_, ^2^σ²_ext_, ^1^V_d,_ ^2^V_d_ and ^2^P_ext_ on performance. Reference conditions used consist of **^1^**σ²_ext_, ^2^σ²_ext_, ^1^V_d,_ ^2^V_d_ values and ^2^P_ext_ set to values obtained with the system *Agilent 1* with UV detection (Table 4).

| **Parameter** | **Condition / Setting** |
| --- | --- |
| **First Dimension** | |
| Column | Waters BEH C18 2.1x20mm 1.7µm |
| Mobile Phase A | 30 mM ammonium hydroxide, pH 9 |
| Mobile Phase B | MeOH 100% |
| Gradient | 3-64 %B, 60 min (equilibration time 2*t_0_) |
| Flow Rate | 150 µL/min |
| Injection Volume | 1.3 µL |
| Analyte | Peptides mixture 0.1 mg/mL in H_2_O/ACN 50:50 (Molecular weight 2000 Da) |
| Temperature | 30 °C |
| **Interface / Modulation** | |
| Sampling Time | 0.22 |
| Sampling Rate | 1.2 cuts/peak |
| Solvent dilution prior to ^2^D | No |
| **Second Dimension** | |
| Column | Waters BEH C18 2.1x30mm 1.7µm |
| Mobile Phase A | 30 mM formic acid, pH 3 |
| Mobile Phase B | ACN 100% |
| Gradient | 5-22%B, 0.10 min (equilibration time 2*t_0_) |
| Flow Rate | 2600 µL/min |
| Temperature | 80 °C |

**Figure S1.** Gradient program and illustration of the determination of t₅₀ used for the measurement of the system dwell volume based on acetone signal during the gradient.


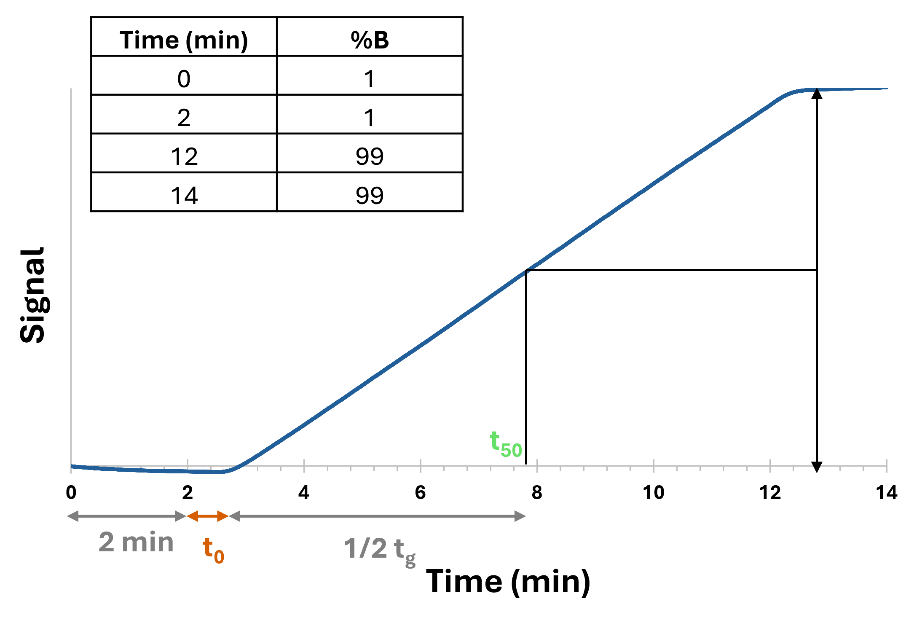


**Figure S2**. Screenshot of the reference conditions defined in the *Optimization* module of the 2D-LC Smart Calculator, used for *in silico* prediction of peak capacity, dilution factor, and run number.


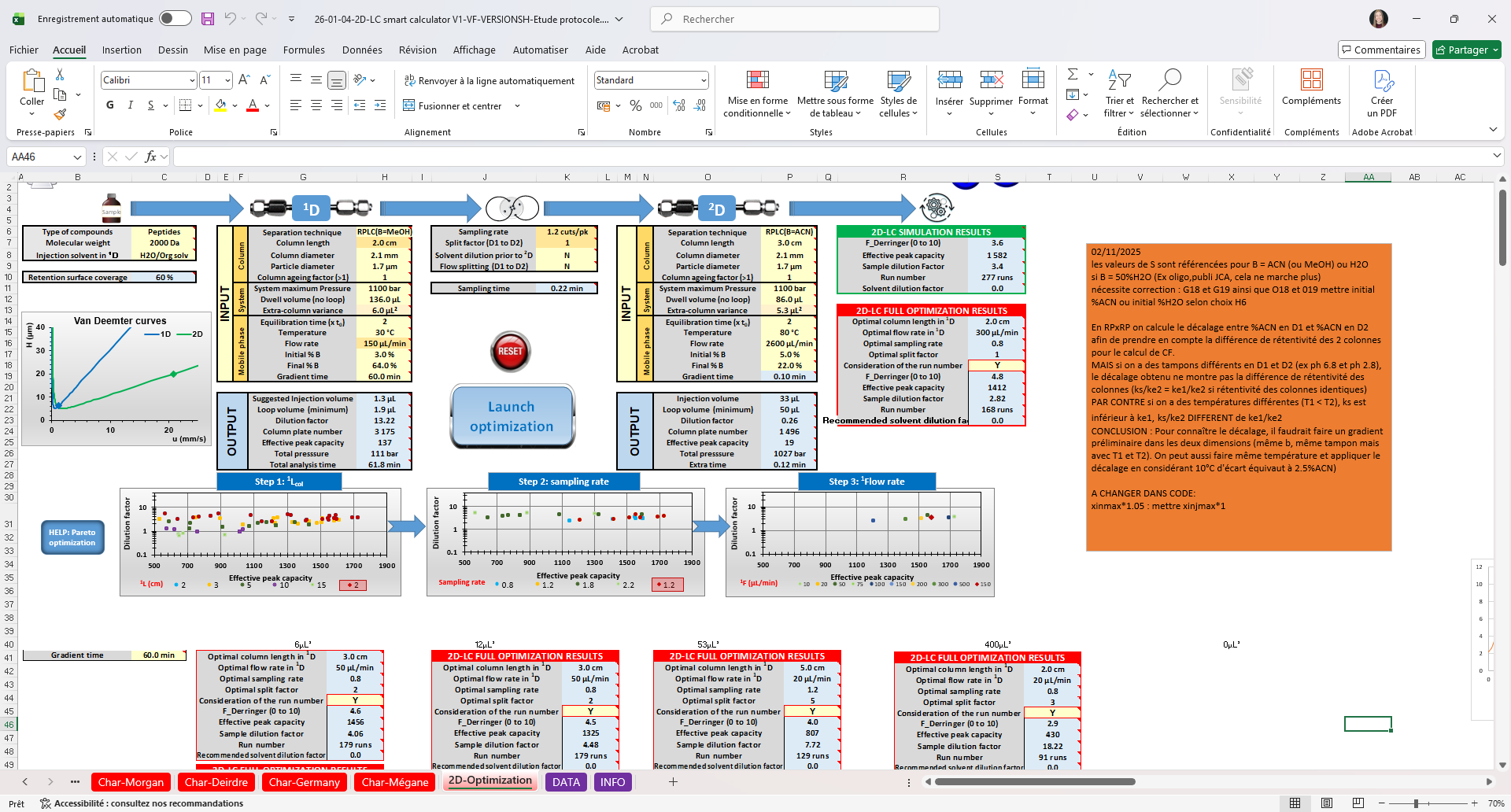

Supplement: Supplementary file 1 — Supporting File: jssc70434‐sup‐0001‐SuppMat.docx. [file JSSC-49-e70434-s001.docx]
